# CCND1, NOP14 and DNMT3B are involved in miR‐502‐5p–mediated inhibition of cell migration and proliferation in bladder cancer

**DOI:** 10.1111/cpr.12751

**Published:** 2020-01-23

**Authors:** Yufan Ying, Jiangfeng Li, Haiyun Xie, Huaqing Yan, Ke Jin, Liujia He, Xueyou Ma, Jian Wu, Xin Xu, Jiajie Fang, Xiao Wang, Xiangyi Zheng, Ben Liu, Liping Xie

**Affiliations:** ^1^ Department of Urology School of Medicine First Affiliated Hospital of Zhejiang University Hangzhou China

**Keywords:** bladder cancer, DNA methylation, metastasis, miRNA, proliferation

## Abstract

**Objectives:**

Downregulation of miR‐502‐5p has emerged as a critical factor in tumour progression in several cancers. Herein, we elucidated the role of miR‐502‐5p in bladder cancer.

**Materials and methods:**

RT‐qPCR was performed to examine the expression of miR‐502‐5p in bladder cancer. And DNA methylation analysis showed that epigenetic mechanisms may contribute to the downregulation of miR‐502‐5p. Then, wound‐healing assay, transwell assay, colony formation assay, CCK8 assay and flow cytometry analysis were applied to evaluate the function of miR‐502‐5p in bladder cancer cell lines. Western blot was conducted to measure the protein levels of related genes. Furthermore, dual‐luciferase reporter assay, in vivo tumorigenesis assay and immunohistochemical staining were also conducted as needed.

**Results:**

MiR‐502‐5p is frequently downregulated in BCa. Meanwhile, hypermethylation of CpG islands contributes to the downregulation of miR‐502‐5p. Functionally, overexpression of miR‐502‐5p inhibited cell proliferation and migration in vitro and repressed tumour growth in vivo. CCND1, DNMT3B and NOP14 were identified as direct targets of miR‐502‐5p. Interestingly, DNMT3B and miR‐502‐5p established a positive feedback loop in the regulation of bladder cancer. In addition, rescue experiments further validated the direct molecular interaction between miR‐502‐5p and its targets.

**Conclusions:**

Our study proposed and demonstrated that the miR‐502‐5p–mediated regulatory network is critical in bladder cancer; this network may be useful in the development of more effective therapies against bladder cancer.

## 1 INTRODUCTION

Bladder cancer (BCa) is the 11th most commonly diagnosed cancer in both genders worldwide and the seventh most commonly diagnosed cancer when only males are considered.^1^ BCa can be classified into muscle‐invasive BCa (MIBC) and non–muscle‐invasive BCa (NMIBC). Approximately one‐quarter of BCa patients develop a disease classed as muscle‐invasive or metastatic.^2^ While various therapeutic strategies have been proposed and used in past decades, radical cystectomy is considered the standard treatment for nonmetastatic MIBC.^3^ Patients with invasive BCa have a low 5‐year survival (62%) after radical cystectomy.^4^ These data support the notion that early diagnoses, personalized treatment programmes and follow‐up care are key factors for a successful outcome.^5^ Continuing efforts to create novel and more effective treatments are needed.

MicroRNAs (miRNAs) belonging to a class of short noncoding RNAs (19‐22 nucleotides) play a significant role in regulating gene expression in numerous cellular processes and human diseases by binding to the 3′ UTR of target mRNAs.^6^ Previous evidence has revealed that abnormal expression of miRNA contributes to the occurrence and development of human BCa.^7^ In addition, we previously confirmed the tumour‐suppressing role of a series of miRNAs, including miR‐22, miR‐148a‐3p, miR‐182‐5p, miR‐320c, miR‐323a‐3p, miR‐409, miR‐433 and miR‐576, which are involved in regulating oncogenicity and progression of BCa.^8^, ^9^, ^10^, ^11^, ^12^, ^13^, ^14^, ^15^ MiR‐502‐5p is located at Xp11.23 and has been reported to be associated with the tumorigenicity and progression of breast cancer,^16^ colon cancer,^17^ liver cancer^18^ and non‐Hodgkin lymphoma.^19^ However, the extract role of miR‐502‐5p in bladder cancer has not been investigated.

In our studies, we revealed that hypermethylation status of chloride voltage‐gated channel 5 (CLCN5) divergent methylation region (DMR) contributed to the downregulation of miR‐502‐5p in bladder cancer. Both in vitro and in vivo studies revealed that miR‐502‐5p could induce G1 phase arrest and cell apoptosis and inhibit EMT progression. Furthermore, CCND1, DNMT3B and NOP14 were identified as downstream targets of miR‐502‐5p. Thus, we introduced a biological mechanism that was induced by miR‐502‐5p and involved in regulating BCa progression.

## 2 MATERIALS AND METHODS

### 2.1 Cell lines and cell culture

We purchased the normal bladder cell line SV‐HUC‐1, the human BCa cell lines T24 and UM‐UC3, and the human embryonic kidney cell line 293T from the Cell Bank of the Chinese Academy of Sciences (Shanghai, China). The 293T cell line was maintained in DMEM (Corning), UM‐UC3 was maintained in MEM (Corning), T24 was maintained in 1640 medium (Corning), and SV‐HUC‐1 was maintained in F‐12K medium (Gibco, Thermo Fisher Scientific) containing 10% heat‐inactivated foetal bovine serum (BI) under a humidified atmosphere of 5% CO_2_ at 37°C.

### 2.2 Human clinical samples

Paired BCa tissues and adjacent nontumorous bladder mucosal tissues were obtained from patients who were treated with BCa radical cystectomy. Ten pairs of samples were collected between January 2011 and October 2013 at the First Affiliated Hospital of Zhejiang University with the informed consent of the patients and approval of the Ethics Committee. Tissue samples were snap‐frozen in liquid nitrogen until RNA extraction.

### 2.3 TCGA database and KEGG pathway analysis

TCGA is available from the Cancer Genomics Browser, University of California, Santa Cruz (http://genome-cancer.ucsc.edu/). KEGG is the Kyoto Encyclopedia of Genes and Genomes (http://www.genome.jp/kegg/). The associated integrated database that we used included the Oncomine (https://www.oncomine.org/resource/login.html), starBase databases (http://starbase.sysu.edu.cn/) and LinkedOmics (http://www.linkedomics.org/login.php), which were used to analyse the expression pattern of CCND1, DNMT3B and NOP14 in BCa tissues. Further potential pathways mediated by miR‐502‐5p were identified by KOBAS (http://kobas.cbi.pku.edu.cn/index.php) by analysing the downstream target genes list predicted by miRWalk, TargetScan and LinkedOmics.

### 2.4 Reagents and transfection

The RNA duplexes were chemically synthesized by GenePharma (Shanghai, China). All of the corresponding sequences were shown in Table S1. To improve the silencing efficiency and avoid off‐target effects, we merged 3 different small interfering RNAs (siRNA) into a siRNA pool to transfect BCa cell lines in our experiments. Oligonucleotide transfections were performed using Lipofectamine 2000 Regent (Thermo Fisher) in accordance with the manufacturer's protocols. Polyplus transfection^®^ reagent (Proteintech, Polyplus Transfection) was used to transfect the constructed plasmid in accordance with the manufacturer's protocols in the rescue experiments and dual‐luciferase reporter assays.

### 2.5 RNA isolation and RT‐qPCR

RNA was extracted from clinical tissues and BCa cell lines with RNAiso Plus (Takara) and transcribed into cDNA by using a One Step PrimeScript miRNA cDNA Synthesis Kit and PrimeScript RT Reagent Kit (Takara). The relative expression levels of mRNA and miRNA were detected by RT‐qPCR, which was performed using the ABI 7500 fast real‐time PCR System (Applied Biosystems) and SYBR Premix Ex Taq (Takara). U6 small nuclear RNA and GAPDH mRNA were used as endogenous references to quantify the relative expression of miRNA and mRNA. All primers used are shown in Table S1.

### 2.6 Dual‐luciferase reporter assay

Oligonucleotide pairs containing the potentially miR‐502‐5p target region (wild‐type) or mutant target region (mutated type) were designed and ordered from Sangon. After the annealing process, these double‐stranded segments were inserted into the pmirGLO Dual‐luciferase miRNA Target Expression Vector (Promega) between the Sac I and Sal I sites. The insertions were validated by DNA sequencing. The 293T cells were seeded in 96‐well plates and cotransfected with 12.5 nmol/L miR‐502‐5p or NC and 25 ng the above‐constructed target reporter pmirGLO. The relative luciferase activity was measured by the dual‐luciferase reporter assay system (Promega) 48 hours after transfection with Berthold Detection System.

### 2.7 DNA methylation analysis

The DNA methylation levels of specific CpG sites of miR‐502‐5p in the promoter region were determined by MethylTarget sequencing (Genesky Biotechnologies Inc), a method using next‐generation sequencing‐based multiple targeted CpG methylation analysis.^20^, ^21^ Primer design and validation were performed by Methylation Primer software on bisulphate‐converted DNA. Primer sets were designed to flank each targeted CpG site in 100‐300 nucleotide regions. Genomic DNA was extracted from frozen samples using Genomic Tip‐500 columns (Qiagen) and from bisulphite‐converted using the EZ DNA Methylation™‐GOLD Kit (Zymo Research) according to the manufacturer's protocols. After PCR amplification (HotStarTaq polymerase kit, TAKARA) and library construction, samples were sequenced (Illumina MiSeq Benchtop Sequencer) using the paired‐end sequencing protocol according to the manufacturer's guidelines.^22^

After bisulphite conversion, the CpG islands of E‐cadherin in the promoter region were amplified by PCR with primers (primer‐F: TTTGTTTTGTTATTTAGGTTGGAG, primer‐R: TAACCAACTTAATAAAACCCCATC). The PCR products were cloned into the pUC18‐T vector. After amplification, 5 clones were sent for DNA sequencing (Sangon).

### 2.8 Cell growth/cell viability assay

T24 and UM‐UC3 cells were seeded in 96‐well plates with 3000 cells per well. After 24 hours of incubation at 37°C, the cells were transfected with the RNA duplex (miR‐502‐5p mimics, NC, si‐NOP14, si‐DNMT3B) at different concentration for 48‐72 hours. Cell viability assays were conducted with the Cell Counting Kit‐8 (Dojindo Laboratories) in accordance with the manufacturer's protocol.

### 2.9 Colony formation assay

The transfected cells were seeded in 6‐well plates (500 cells per well) and maintained under standardized culture conditions for 14 days. Colony counts and rates were calculated after the colonies were fixed with absolute methanol and stained with 0.1% crystal violet.

### 2.10 Cell cycle analysis

After digestion and washing in PBS, transfected cells were fixed with 70% ethanol overnight at 4°C. Cell cycle assays were performed with the BD LSRII Flow Cytometer System with FACSDiva software (BD Biosciences). The data were analysed by ModFit LT 3.2 software (Verity Software House). The detailed steps and procedures have been previously described.^15^

### 2.11 Western blot assay

Western blot assays were conducted as previously described.^8^ The associated primary immunoblotting antibodies were as follows: anti‐GAPDH, anti–E‐cadherin, anti–N‐cadherin, anti‐Vimentin, anti‐CDK6, anti‐CCND1, anti‐CDK4, anti‐NOP14, anti‐MMP9, anti‐MMP2, anti‐E2F1, anti‐MET, anti‐Parp‐1, (Proteintech Group), anti‐Caspase 3, anti‐DNMT3B (Cell Signaling Technology) and anti‐SP1 (Santa Cruz Biotechnology).

### 2.12 Transwell assay

Migration ability was measured by transwell assay with transwell chambers (Millipore). After transfection, 4 × 10^4^ T24 cells and 7 × 10^4^ UM‐UC3 cells were suspended in 200 µL of serum‐free medium and added to the chambers. The chambers were placed into a 24‐well plate, and 800 µL RPMI‐1640 or MEM containing 10% foetal bovine serum was added to the space between the chamber and the well. After incubation for 24 hours at 37°C, we detected the migration rates using methanol and 0.1% crystal violet. The photographs were obtained under phase‐contrast microscopy (Carl Zeiss) with a 4 × and 20 × objective lens.

### 2.13 Wound‐healing assay

After transfection, the cells were grown to 100% confluence in 6‐well plates. A micropipette tip was used to make a cross‐wound, and wound healing was observed after 24 hours of standard culture. Photographs were obtained under phase‐contrast microscopy (Olympus) with a 4× objective.

### 2.14 Cell apoptosis analysis

After digestion and washing with PBS, transfected cells were resuspended in 1× binding buffer. Then, 5 μL Annexin V‐FITC and 10 μL PI were added to each sample, and the mixture was incubated in the dark at room temperature for 5 minutes. Cell apoptosis assays were performed with the BD LSRII Flow Cytometer System with FACSDiva software (BD Biosciences). The data were analysed by FACSDiva software.

### 2.15 In vivo tumorigenesis assay

Ten male BALB/c‐nude mice (4‐week‐old) were ordered from the Shanghai Experimental Animal Center (Chinese Academy of Sciences). Lentivirus‐transfected UM‐UC3 cells (1.5 × 10^6^ cells per mouse) that stably expressed firefly luciferase were injected subcutaneously into the flanks of mice. Thirty micrograms of Lipofectamine 2000‐encapsulated NC or miR‐502‐5p was used for injection when palpable tumours arose. The size of the tumour [*V* = (width^2^ × length × 0.52)] was measured by a Vernier caliper every 3 days. After 4 weeks, bioluminescence of the tumour was detected by an in vivo bioluminescence imaging system. Then, the mice were sacrificed, and the tumours were measured. The tumour specimens were embedded in paraffin for IHC analysis. All animal studies and manipulation were performed according to the institutional guidelines provided by the First Affiliated Hospital, School of Medicine, Zhejiang University.

### 2.16 Immunohistochemical staining

Tumour graft samples anatomized from mice were analysed by immunohistochemical staining. The associated antibodies are as follows: anti‐Ki‐67, anti‐CCND1, anti‐NOP14 (Proteintech) and anti‐DNMT3B (Cell Signaling Technology). Antigen retrieval was performed by heating the slides in sodium citrate buffer (10 mmol/L, pH 6.0). After blocking with bovine serum albumin (Sango Biotech, Shanghai, China), the slides were incubated with anti‐Ki‐67, anti‐CCND1, anti‐NOP14 (Proteintech) and anti‐DNMT3B (Cell Signaling Technology) overnight at 4°C. The slides were then incubated with the secondary antibody goat anti‐rabbit HRP (Cell Signaling Technology) conjugate for 1 hours at room temperature. A DAB solution was used for brown colour development. The strength of positivity was used to semiquantify the strength of positivity, which considered the intensity of the staining and the percentage of positive cells per the formula.

### 2.17 In situ hybridization (ISH)

A 5′‐DIG and 3′‐DIG‐labelled, locked nucleic acid‐incorporated miRNA probe (miRCURY LNA™ Detection probe, Exiqon) was used for the visualization of miR‐502‐5p in the same bladder cancer samples. Paraffin tissue slides were deparaffinized and digested with proteinase K for 6.5 min (15 μg/mL). The slides were then prehybridized in a hybridization solution at 50°C for 1 hours. Tissues were hybridized for 2 days in the presence of 10 ng 3′‐5′ DIG‐labelled miR‐148a LNA probes at 4°C (500 nmol/L). Slides were washed stringently for 20 minutes at 50°C, and an immunological reaction was conducted using anti–DIG‐AP Fab fragments according to the manufacturer's protocol. The strength of positivity was semiquantified by considering both the intensity and proportion of positive cells.

### 2.18 Statistical analysis

The data are expressed as the means ± SD. Differences between groups were estimated by Student's *t* test or chi‐square test. All analyses were performed by SPSS 16.0 (IBM), and statistical significance was defined as a two‐tailed value of *P* < .05.

## 3 RESULTS

### 3.1 MiR‐502‐5p is frequently downregulated in BCa

**Figure 1:**
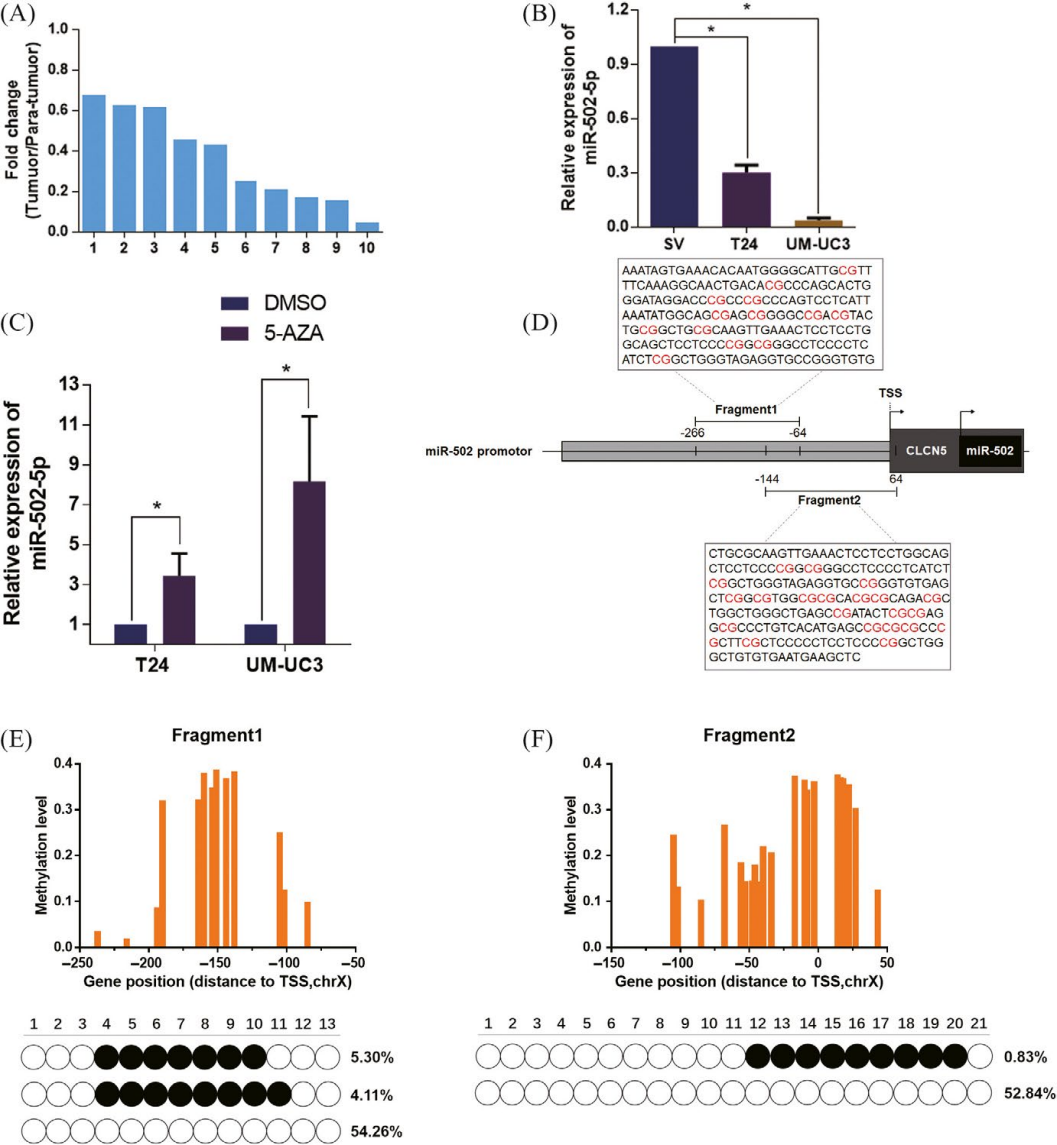
MiR‐502‐5p is frequently downregulated in BCa. A, Relative expression levels of miR‐502‐5p in 10 pairs of BCa tissues are shown by comparing the corresponding adjacent normal tissues. B, Relative expression levels of miR‐502‐5p in BCa cell lines (T24 and UM‐UC3) compared with those in normal cell lines (SV‐HUC‐1). C, The expression of miR‐502‐5p was upregulated after the treatment of demethylating agent 5‐aza‐dC. D, Schematic diagram showed the promoter region of miR‐502‐5p. CpG islands, determined in this study, on 5′‐flanking promoter regions of miR‐502‐5p localized between −266 and 64 bp relative to the transcription start site (TSS). E, Methylation rate on promoter from −266 to −64 bp in T24 cell lines, and the top 3 haplotypes of high frequency are shown. F, Methylation rate on promoter from −144 to 64 bp in T24 cell lines, and the top 2 haplotypes of high frequency are shown. *P < .05

To examine the miR‐502‐5p level in bladder cancer, we initially performed an RT‐qPCR assay to analyse the expression pattern of miR‐502‐5p in 10 pairs of clinical BCa tissues and adjacent noncancerous tissues (clinical characteristics of the patients are presented in Table S2). The results indicated a significant reduction in miR‐502‐5p levels in BCa tissues (Figure 1A). In addition, ISH analysis demonstrated that miR‐502‐5p expression was significantly downregulated in bladder cancer tissues compared with adjacent non‐tumour tissues (Figure S4E,F). Consistently, the examination of miR‐502‐5p in T24 and UM‐UC3 cell lines showed significant downregulation compared with the SV‐HUC‐1 cell line (Figure 1B).

These results demonstrated that miR‐502‐5p may play a potential regulatory role in BCa. MiR‐502‐5p is located at chromosome Xp11.23 and belongs to the CLCN5 region and numerous miRNAs of which have been confirmed to involve in divergent types of tumours. Previous studies indicated several miRNAs were downregulated in tumours due to the hypermethylated status of CpG islands in the promoter region.^13^, ^14^ To evaluate the methylation status of CLCN5 and the regulatory impact on miR‐502‐5p in BCa, RT‐qPCR was performed to demonstrate the expression changes of miR‐502‐5p in T24 and UM‐UC3 cell lines after 5‐aza‐CdR treatment. Results indicated a significant upregulation of miR‐502‐5p in BCa cell lines treated with 5‐aza‐CdR (Figure 1C). Furthermore, MethylTarget sequencing assay was performed to test the CpG island methylation level of miR‐502‐5p in the promoter region in T24 cell line. And two regions of CpG islands were analysed (Figure 1D). Results indicated the promoter CpG hypermethylation might contribute to the dysregulation of miR‐502‐5p in BCa (Figure 1E,F). Thus, results demonstrated that miR‐502‐5p is downregulated in BCa due to the hypermethylation of CpG islands, and miR‐502‐5p may play a tumour‐suppressing role in BCa.

### 3.2 Overexpression of miR‐502‐5p inhibits cell proliferation and migration of BCa cell lines in vitro

**Figure 2:**
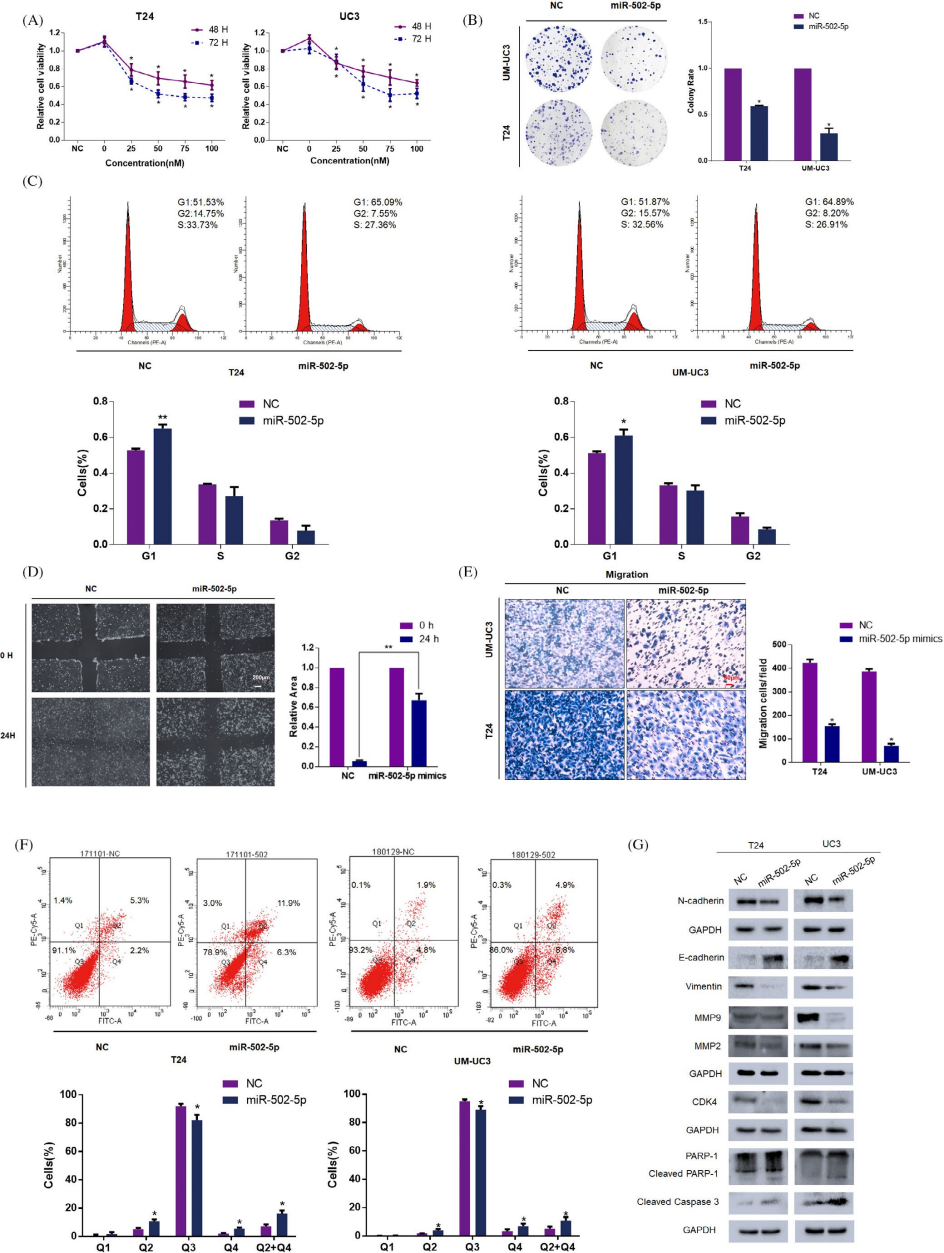
Overexpression of miR‐502‐5p inhibits the proliferation and migration of BCa cells. A, CCK8 assay. The relative cell viabilities of T24 and UM‐UC3 after exposure to different concentrations of the miR‐502‐5p mimic were notably lower than those in the NC‐treated groups at 48 and 72 h. B, Colony formation assay (representative wells are presented). The colony formation rate was lower for the miR‐502‐5p mimic‐treated group than for the NC‐treated group. C, Cell cycle assay (representative histograms are presented). Overexpression of miR‐502‐5p notably induced G1 phase arrest in T24 and UM‐UC3 cell lines. D, Wound‐healing assay. The wound‐healing efficacy of the miR‐502‐5p mimic (50 nmol/L)‐transfected group was retarded at 24 h in the T24 cell line. E, Transwell assay (representative micrographs are presented). Migration rates were reduced by the overexpression of miR‐502‐5p. F, Cell apoptosis assay (representative histograms are presented). Overexpression of miR‐502‐5p significantly induced cell apoptosis in the T24 and UM‐UC3 cell lines. G, Western blot assay. Noticeable inhibition of EMT, MMPs, cell apoptosis and cell cycle‐associated proteins were detected by the overexpression of miR‐502‐5p in BCa cells. Error bars represent the SE obtained from 3 independent experiments; *P < .05

To investigate the tumour inhibition effect of miR‐502‐5p in BCa cell lines, T24 and UM‐UC3 cell lines were transfected with miR‐502‐5p mimics for 48 or 72 hours Cell viability was determined by CCK8 assay, and the results revealed the suppression of cell viability at different concentrations and time points (Figure 2A). Simultaneously, colony formation assay revealed that miR‐502‐5p could diminish the colony rate in BCa cell lines (Figure 2B). To examine the underlying mechanisms of proliferation inhibition, we performed flow cytometry assay. We observed obvious G1 phase arrest and apoptosis induced by the forced expression of miR‐502‐5p in BCa cell lines (Figure 2C). Consistently, the significant inhibition of the G1/S transition regulators CDK4 was also confirmed by Western blot assay (Figure 2G).

In addition, flow cytometry analysis indicated that miR‐502‐5p markedly promoted apoptosis compared with the control in T24 and UM‐UC3 cell lines (Figure 2F). Furthermore, miR‐502‐5p upregulation led to the increased expression of apoptosis‐related proteins including Caspase 3 and Parp‐1 (Figure 2G). Consequently, the overexpression of miR‐502‐5p significantly suppressed cell proliferation in BCa cells via G1 phase arrest and cell apoptosis.

A wound‐healing assay was performed to examine whether miR‐502‐5p had any effect on the migration ability of BCa. We observed delayed wound closure in the miR‐502‐5p transfected group compared with the control group (Figure 2D). In addition, transwell assays indicated that the overexpression of miR‐502‐5p significantly suppressed cell migration in both cell lines (Figure 2E). Consistently, miR‐502‐5p overexpression of inhibited the EMT pathway and MMP2 and MMP9 proteins. These results demonstrated that the overexpression of miR‐502‐5p significantly suppressed the proliferation and migration of BCa cell lines. Accordingly, miR‐502‐5p overexpression markedly repressed the migration of BCa in vitro.

### 3.3 CCND1, DNMT3B and NOP14 are direct targets of miR‐502‐5p

**Figure 3:**
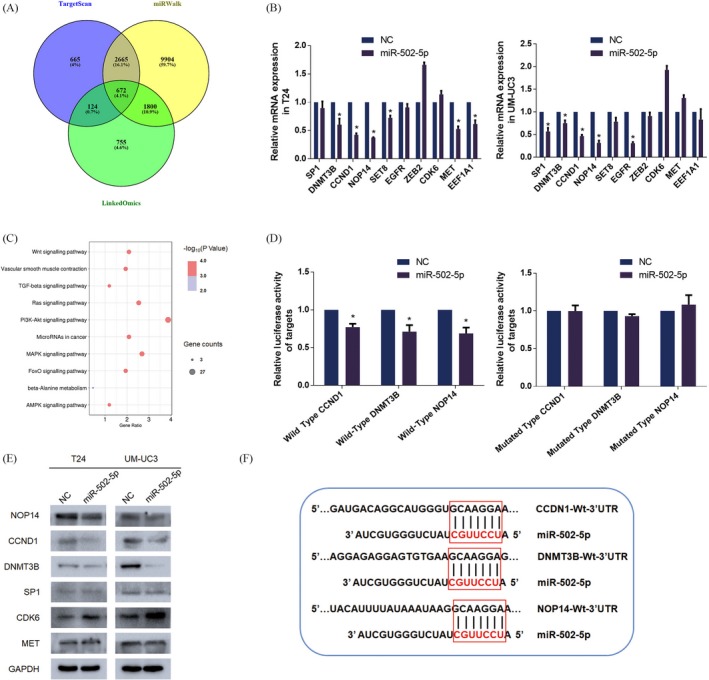
CCND1, DNMT3B and NOP14 were identified as direct downstream targets of miR‐502‐5p. A, Bioinformatic prediction analysis. MiRWalk and TargetScan online databases were used to predict potential downstream targets of miR‐502‐5p. B, RT‐qPCR assay. Ten candidate targets were selected, and the mRNA levels of these genes were measured. Only CCND1, DNMT3B and NOP14 were significantly downregulated in both cell lines. C, KEGG pathways analysis. KOBAS tool was used to analyse the pathways involved (representative pathways are presented). D, Dual‐luciferase reporter assay. The luciferase activity was significantly reduced after miR‐502‐5p mimics were transfected into the wild‐type group. However, no marked changes in the luciferase activity were observed in the mutated type group. E, Western blot assay. Protein levels of several candidate targets were measured after transfection with miR‐502‐5p mimics. F, Schematic diagram of the miR‐502‐5p–targeting region of CCND1, DNMT3B and NOP14 with seed matching. Error bars represent the SE obtained from three independent experiments; *P < .05

To identify direct targets of miR‐502‐5p, we initially used bioinformatics prediction websites (TargetScan, LinkedOmics and miRWalk) for primary screening analysis.^23^, ^24^, ^25^ A total of 672 genes were found in all three databases (Figure 3A). Furthermore, pathway analysis of the 672 genes was performed with KOBAS 3.0.^26^ The results demonstrated that 100 signal pathways were predicted to be involved (*P* < .05), most of which associated with cancers or signal pathways, including PI3K‐AKT signalling pathway (Table 1, Figure 3C). Considering the common targets that were predicted by the databases, several candidate target genes with relatively high scores were chosen and preliminarily verified by RT‐qPCR. We observed that the downregulation of 3 mRNAs (CCND1, DNMT3B and NOP14) was induced by miR‐502‐5p (Figure 3B). Western blot assays showed a consistent reduction in expression at the protein level after miR‐502‐5p mimic treatment (Figure 3E).

**Table 1:** KEGG pathways analysis (*P* < .05) of 672 common potential targets with KOBAS tool

Signalling pathway	Gene counts (n)	*P*‐value
Pathways in cancer	35	1.91E−14
Axon guidance	20	1.36E−10
PI3K‐Akt signalling pathway	26	8.65E−10
cGMP‐PKG signalling pathway	17	1.47E−08
Regulation of actin cytoskeleton	19	1.76E−08
Hippo signalling pathway	16	3.00E−08
Renin secretion	11	4.61E−08
HTLV‐I infection	20	5.97E−08
Focal adhesion	17	2.01E−07
Hedgehog signalling pathway	9	3.46E−07
Vascular smooth muscle contraction	13	3.79E−07
Wnt signalling pathway	14	4.23E−07
Ras signalling pathway	17	9.12E−07
MAPK signalling pathway	18	9.21E−07
Melanoma	10	9.79E−07
Proteoglycans in cancer	16	1.09E−06
FoxO signalling pathway	13	1.20E−06
Adherens junction	10	1.38E−06
Rap1 signalling pathway	16	1.55E−06
Colorectal cancer	9	2.73E−06
Metabolic pathways	45	3.03E−06
Adrenergic signalling in cardiomyocytes	13	3.55E−06
Calcium signalling pathway	14	5.25E−06
Gap junction	10	5.67E−06
cAMP signalling pathway	14	1.52E−05
Endocytosis	16	1.84E−05
Longevity regulating pathway—multiple species	8	2.62E−05
Insulin secretion	9	2.78E−05
Pancreatic cancer	8	3.21E−05
Cholinergic synapse	10	3.64E−05
Salivary secretion	9	3.88E−05
Dilated cardiomyopathy	9	4.20E−05
Signalling pathways regulating pluripotency of stem cells	11	5.46E−05
Longevity regulating pathway	9	5.75E−05
Phospholipase D signalling pathway	11	6.15E−05
Chronic myeloid leukaemia	8	6.18E−05
Pancreatic secretion	9	6.68E−05
Gastric acid secretion	8	6.75E−05
Phosphatidylinositol signalling system	9	7.74E−05
Oocyte meiosis	10	8.13E−05
Regulation of lipolysis in adipocytes	7	8.39E−05
Melanogenesis	9	8.94E−05
Chagas disease (American trypanosomiasis)	9	1.18E−04
Dopaminergic synapse	10	1.25E−04
ECM‐receptor interaction	8	1.31E−04
Oxytocin signalling pathway	11	1.33E−04
TGF‐beta signalling pathway	8	1.53E−04
Glioma	7	1.96E−04
GABAergic synapse	8	2.05E−04
Morphine addiction	8	2.54E−04
Notch signalling pathway	6	2.70E−04
Circadian entrainment	8	3.32E−04
Transcriptional misregulation in cancer	11	3.84E−04
Inflammatory mediator regulation of TRP channels	8	4.04E−04
Oestrogen signalling pathway	8	4.30E−04
Basal cell carcinoma	6	5.27E−04
Aldosterone synthesis and secretion	7	6.74E−04
MicroRNAs in cancer	14	8.51E−04
Serotonergic synapse	8	9.19E−04
Small cell lung cancer	7	9.36E−04
Glutamatergic synapse	8	1.02E−03
Viral carcinogenesis	11	1.06E−03
Purine metabolism	10	1.18E−03
Thyroid hormone signalling pathway	8	1.26E−03
GnRH signalling pathway	7	1.27E−03
Neurotrophin signalling pathway	8	1.39E−03
Platelet activation	8	1.54E−03
AMPK signalling pathway	8	1.78E−03
Thyroid hormone synthesis	6	1.80E−03
Endocrine resistance	7	1.80E−03
Type II diabetes mellitus	5	1.86E−03
Progesterone‐mediated oocyte maturation	7	1.90E−03
Amoebiasis	7	2.11E−03
Arrhythmogenic right ventricular cardiomyopathy (ARVC)	6	2.19E−03
AGE‐RAGE signalling pathway in diabetic complications	7	2.23E−03
Choline metabolism in cancer	7	2.23E−03
Cytokine‐cytokine receptor interaction	12	2.53E−03
Mucin type O‐Glycan biosynthesis	4	2.63E−03
Glycosphingolipid biosynthesis—ganglio series	3	3.10E−03
Hypertrophic cardiomyopathy (HCM)	6	3.73E−03
Prostate cancer	6	5.14E−03
Lysosome	7	6.19E−03
Glycosaminoglycan biosynthesis—chondroitin sulphate/ dermatan sulphate	3	6.32E−03
Long‐term potentiation	5	6.60E−03
Amphetamine addiction	5	7.00E−03
Central carbon metabolism in cancer	5	7.00E−03
Renal cell carcinoma	5	7.00E−03
Inositol phosphate metabolism	5	8.76E−03
Retrograde endocannabinoid signalling	6	9.06E−03
Ubiquitin mediated proteolysis	7	1.06E−02
Tight junction	7	1.14E−02
Ovarian steroidogenesis	4	1.24E−02
Complement and coagulation cascades	5	1.32E−02
Dorso‐ventral axis formation	3	1.45E−02
Thyroid cancer	3	1.59E−02
Pentose phosphate pathway	3	1.59E−02
Toxoplasmosis	6	1.84E−02
beta‐Alanine metabolism	3	1.87E−02
Circadian rhythm	3	1.87E−02
Acute myeloid leukaemia	4	1.87E−02

To further verify whether miR‐502‐5p directly interacted with the 3′ UTRs of CCND1, DNMT3B and NOP14, we performed a dual‐luciferase reporter assay. The 3′ UTRs of three genes were cloned into the pmirGLO vector, and we performed a dual‐luciferase reporter assay. We observed a marked decrease in luciferase activity in the wild‐type 3′ UTRs of CCND1, DNMT3B and NOP14 after transfection of miR‐502‐5p. As expected, there was no significant variation in the 3′ UTRs of the mutated groups (Figure 3D). A schematic diagram of the targeting sequence was presented (Figure 3F). In conclusion, these data indicated that CCND1, DNMT3B and NOP14 were the direct downstream targets of miR‐502‐5p.

### 3.4 Silencing of NOP14 induces cell cycle arrest and cell apoptosis in vitro

The role of NOP14 in bladder cancer is still unclear. We used the starBase database to analyse NOP14 expression in 167 BCa patients and 10 patients without BCa for clinical validation.^24^ The statistical analysis revealed a marked upregulation in NOP14 in BCa patients compared to patients without BCa (Figure S1B). However, no marked overall survival difference was detected, which may result from insufficient clinical samples (Figure S1D). The results revealed that NOP14 may play an important role in the progression of BCa.

**Figure 4:**
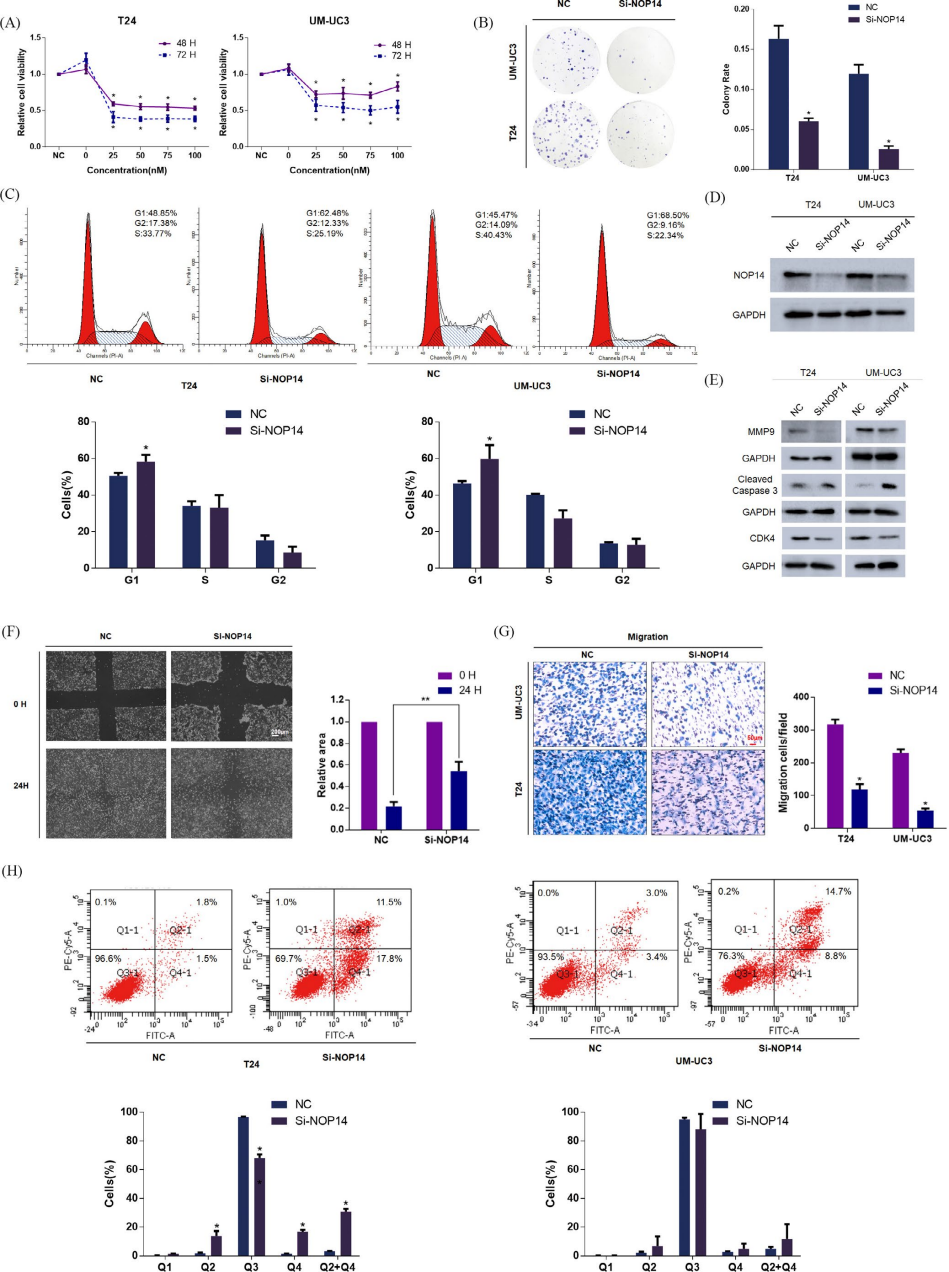
Silencing of NOP14 inhibits proliferation and migration in T24 and UM‐UC3 cell lines. A, CCK8 assay. The NOP14 siRNA pool markedly reduced the cell viability of BCa cells at different concentrations. B, Colony formation assay (representative wells are presented). The colony rate of the Si‐NOP14–transfected group was significantly reduced compared to that of the NC‐transfected group. C, Cell cycle assay (representative histograms are presented). Silencing of NOP14 markedly induced G1 phase arrest in T24 and UM‐UC3 cell lines. D, Western blot assay. Three NOP14 siRNAs were merged into an siRNA pool for higher interference efficiency of NOP14. The protein levels of NOP14 were presented. E, Western blot assay. MMP9, cell apoptosis and cell cycle‐associated proteins were inhibited after silencing NOP14. F, Wound‐healing assay. Silencing of NOP14 retarded the healing efficacy of T24 cells at 24 h. G, Transwell assay (representative micrographs are presented). Silencing of NOP14 significantly inhibited the migration of BCa cells. H, Cell apoptosis assay (representative histograms presented). Silencing of NOP14 significantly induced cell apoptosis in T24 and UM‐UC3 cell lines. Error bars represent the SE obtained from three independent experiments; *P < .05

Three different NOP14 siRNAs were merged into a siRNA pool to avoid off‐target effects. We initially analysed the effect of NOP14 on cell proliferation. The CCK8 assay showed a significant reduction in cell viabilities in the si‐NOP14 group compared with the NC group at different concentration levels (25, 50, 75, 100 nmol/L; Figure 4A). Consistently, cell colony growth rates were also repressed by knockdown of NOP14 in T24, UM‐UC3 cell lines (Figure 4B). We observed G1 phase arrest in both cell lines after si‐NOP14 transfection (Figure 4C). The knockdown efficiency of siRNAs was confirmed by Western blot assay and RT‐qPCR (Figures 4D and S1H). With respect to migration ability, the results of wound‐healing and transwell assays revealed that knockdown of NOP14 markedly suppressed the migration in BCa cells (Figure 4F,G). In addition, silencing NOP14 significantly induced cell apoptosis in T24 and UM‐UC3 cell lines (Figure 4H). As expected, downregulation of NOP14 led to an obvious increase in cleaved Caspase 3. Additionally, knockdown of NOP14 induced a significant reduction of CDK4 and MMP9 (Figure 4E). All the results indicated that NOP14 could regulate BCa cell migration and proliferation.

### 3.5 Silencing of DNMT3B inhibits cell proliferation and migration of BCa cells in vitro

**Figure 5:**
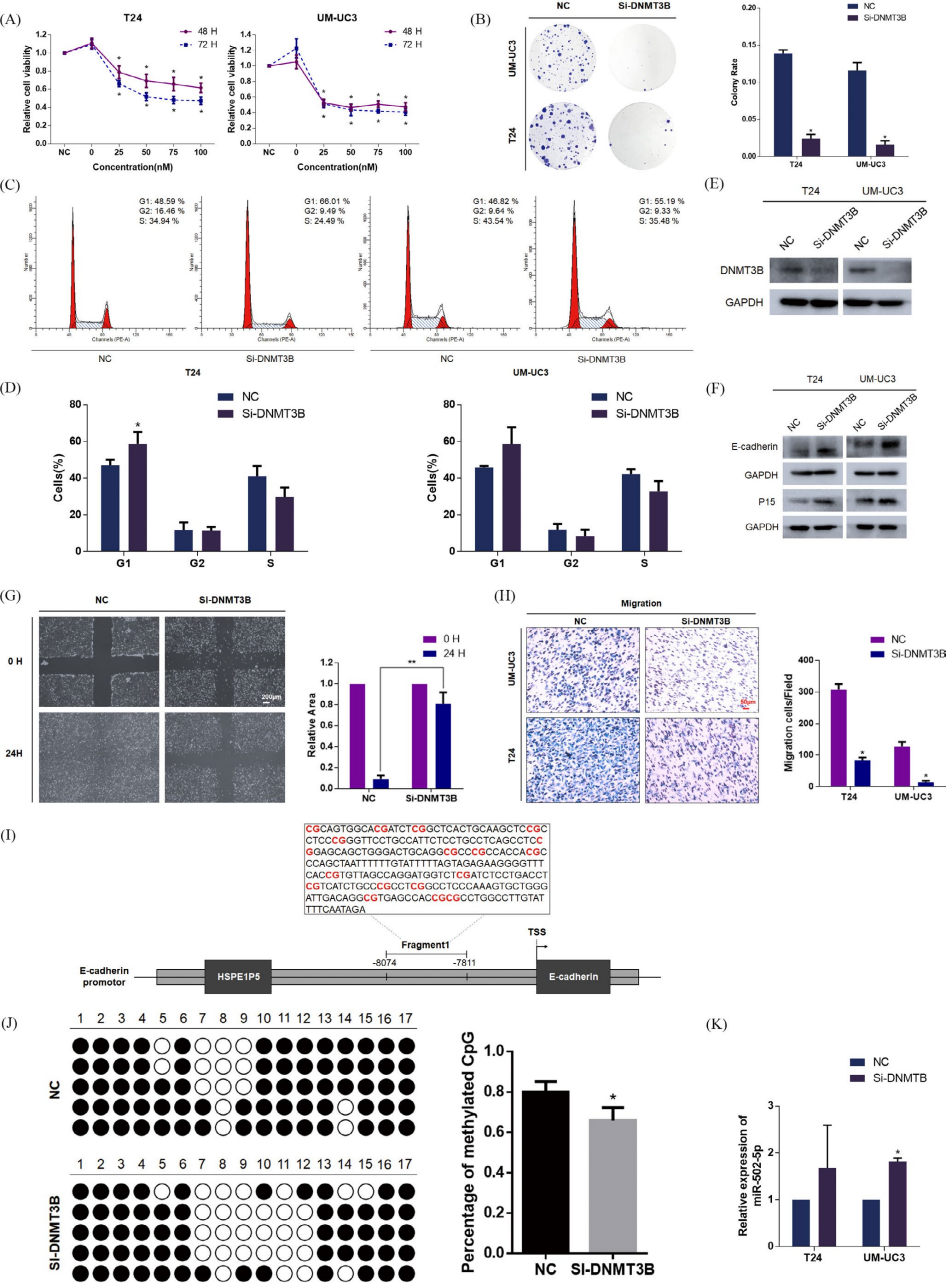
Silencing of DNMT3B inhibits the cell migration and proliferation of T24 and UM‐UC3 cell lines. A, CCK8 assay. Silencing of DNMT3B significantly inhibited cell viability. B, Colony formation assay (representative wells are presented). The colony rate of the si‐DNMT3B transfected group was significantly reduced compared with the NC‐treated group. C and D, Cell cycle assay (representative histograms are presented). Treatment of the siRNA pool in DNMT3B markedly induced G1 phase arrest both in T24 and UM‐UC3 cell lines. E, Western blot assay. Three DNMT3B siRNAs were merged into the siRNA pool to obtain higher interference efficiency. F, Western blot assay. Si‐DNMT3B inhibited EMT and cell cycle‐related proteins. G, Wound‐healing assay. Silencing of DNMT3B retarded the healing of T24 cells at 24 h. H, Transwell assay (representative micrographs are presented). si‐DNMT3B impaired migration of T24 and UM‐UC3 cells. I, Schematic diagram showed the promoter region of E‐cadherin. J, Methylation status of each CpG island detected and calculated by bisulphite‐sequencing PCR experiment. And percentage of methylated CpG islands was performed. K, The expression of miR‐502‐5p was upregulated after silencing DNMT3B. Error bars represent the SE obtained from three independent experiments; *P < .05; **P < .01; ***P < .001

The ONCOMINE database was used to analyse DNMT3B expression in 81 patients with Infiltrating Bladder Urothelial Carcinoma, 28 patients with superficial bladder cancer and 48 patients without bladder cancer.^27^ Results demonstrated a significant upregulation in DNMT3B in bladder cancer compared with normal persons. (Figure S3F). In our study, we merged three individual DNMT3B siRNAs into a siRNA pool to avoid off‐target effects. A CCK8 assay was performed to evaluate the effect of DNMT3B on cell proliferation. The results showed that knockdown of DNMT3B by si‐DNMT3B significantly inhibited the growth of BCa cells at different concentrations and time points (48 and 72 hours; Figure 5A). Coincidentally, colony formation was restrained by knockdown of DNMT3B (Figure 5B). In addition, we detected G1 phase arrest in the T24 and UM‐UC3 cell lines by silencing DNMT3B (Figure 5C,D). The efficiency of si‐DNMT3B on cell migration ability was evaluated by wound‐healing assay and transwell assay, which revealed that DNMT3B silencing could inhibit the migration of BCa cells (Figure 5G,H). The protein levels of the si‐DNMT3B–transfected and NC‐transfected groups are presented (Figure 5E). The Western blot assay showed that knockdown of DNMT3B resulted in a marked upregulation of E‐cadherin and p15 (Figure 5F).

As a member of the DNA methyltransferase family, DNMT3B is thought to function in de novo methylation. E‐cadherin, as a tumour suppressor gene, may be regulated by epigenetic silencing associated with hypermethylation. We next used the NovoPro (http://www.novopro.cn/tools/cpg_islands.html) to identify a CpG island 0‐9000 bp upstream of the E‐cadherin TSS (Figure 5I). Furthermore, bisulphite‐sequencing PCR assay was conducted to assess the methylation level changes of the CpG island by silencing DNMT3B (Figure 5J). As expected, silencing DNMT3B could also upregulate the expression of miR‐502‐5p, which established a positive feedback loop to regulate the progression of BCa (Figure 5K). As expected, treatment of 5‐aza‐CdR significantly inhibited cell migration and proliferation in T24 and UM‐UC3 cell lines (Figure S4A,C), which was evaluated by transwell assay and colony formation assay. In addition, the Western blot assay showed that 5‐aza‐CdR resulted in a significant upregulation of E‐cadherin and p15 (Figure S4B). In summary, DNMT3B plays a crucial role in regulating the progression of BCa.

### 3.6 NOP14 and DNMT3B abrogate the oncogenic effect of miR‐502‐5p in BCa cells

Furthermore, rescue experiments were performed to demonstrate the interactions between miR‐502‐5p and NOP14. The miR‐502‐5p inhibitor partially abrogated the inhibition of cell migration and proliferation abilities induced by NOP14 silencing (Figure S1A,C). Consistently, the elevated cell apoptosis ability was partly abrogated by the miRNA inhibitor (Figure S1E,F). In addition, the overexpression of NOP14 partially rescued the inhibition of cell proliferation, migration ability and G1 phase arrest induced by miR‐502‐5p mimics (Figure S2A‐F). Moreover, the effect of the rescue experiments on cell apoptosis was also evaluated by flow cytometry analysis, which demonstrated that the apoptosis ability promoted by miR‐502‐5p could be restored by NOP14 overexpression (Figure S2G,H). The protein levels of NOP14 in the rescue experiments are presented (Figures S1G and S2I).

Moreover, rescue experiments were performed by cotransfecting miR‐502‐5p mimics and si‐DNMT3B in BCa cells to estimate the interactions between miR‐502‐5p and DNMT3B. We found that the colony formation and migration abilities of BCa cells cotransfected with miR‐502‐5p mimics and empty plasmid were reduced compared with that of cells cotransfected with miR‐502‐5p mimics and plasmid that carried the DNMT3B gene (Figure S3A,B,D). The results demonstrated that DNMT3B upregulation could partly abolish the inhibition of proliferation and migration induced by miR‐502‐5p. Similarly, the overexpression of DNMT3B could also partly attenuate miR‐502‐5p–induced G1 phase arrest (Figure S3C). Additionally, the protein levels of DNMT3B in the rescue experiments are presented (Figure S3E). Collectively, these results demonstrated that miR‐502‐5p inhibited BCa cell progression partly by impairing the oncogenic role of NOP14 and DNMT3B.

### 3.7 CCND1 acts as a target of miR‐502‐5p in regulating the cell cycle of BCa cells

Previous work has shown that CCND1 plays a key role in regulating the cell cycle of BCa cells,^9^ and downregulation of CCND1 induced G1 phase arrest and the inhibition of cell proliferation in BCa. The TCGA data integrated and analysed by the ONCOMINE database revealed that the expression of CCND1 was markedly reduced in bladder cancer (Figure S3G). Consequently, CCND1, targeted by miR‐502‐5p, can regulate cell proliferation in BCa cells.

### 3.8 Tumour suppressor role of miR‐502‐5p in vivo

**Figure 6:**
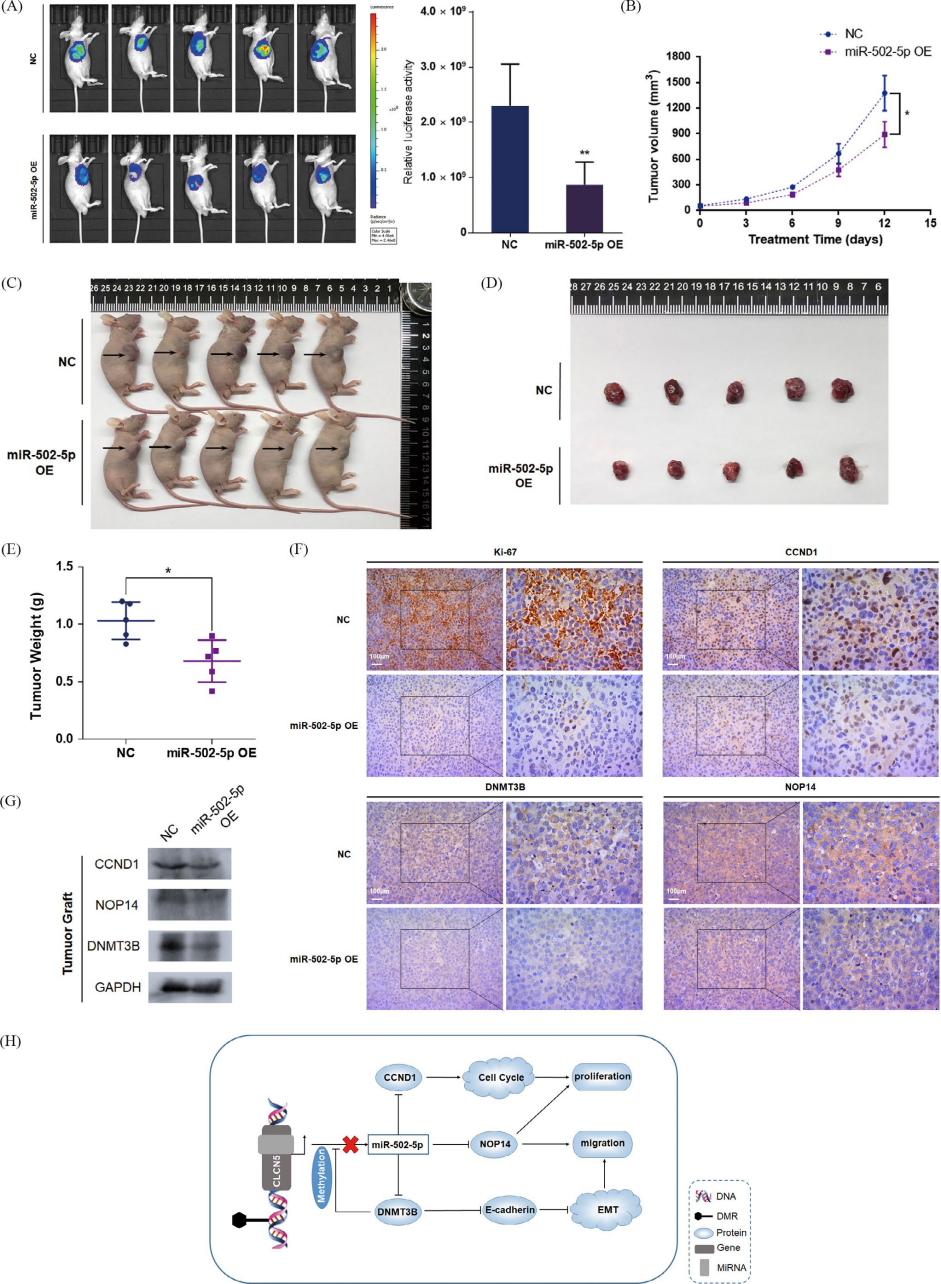
Tumour‐suppressing role of miR‐502‐5p in vivo. A, Fluorescent microscopic image analysis. The luciferase activity of the miR‐502‐5p–transfected group was significantly reduced compared with the NC group. B‐E, Tumour xenograft model. Tumour volumes and growth curves revealed that the overexpression of miR‐502‐5p significantly suppressed tumour growth in vivo. F, Immunohistochemistry results (representative images are presented). Ki‐67 and three targets of miR‐502‐5p were evaluated. G, Western blot assay. Proteins extracted from a tumour graft in each group indicated that three targets of miR‐502‐5p were downregulated in the miR‐502‐5p–treated group. H, Schematic diagram indicated that miR‐502‐5p–mediated regulatory network regulated BCa proliferation and migration. Error bars represent the SE obtained from three independent experiments; *P < .05; **P < .01

All of the above in vitro studies revealed that miR‐502‐5p could promote cell apoptosis and inhibit the migration and proliferation of BCa cells by targeting CCND1, DNMT3B and NOP14. Furthermore, we established a tumour xenograft in ten BALB/c‐nude mice burdened with the UM‐UC3 cell line to examine the role of miR‐502‐5p in vivo. We recorded the tumour growth size of the miR‐502‐5p–treated group and NC‐treated group, which revealed the subcutaneous tumour growth was delayed after transfection with miR‐502‐5p mimics (Figure 6A,B). After 2 weeks, the relative luciferase activities of mice were measured, which indicated a significant reduction in the miR‐502‐5p in the treated group compared with that in the NC‐treated group (Figure 6C). Then, the mice were sacrificed, and the tumour xenografts were weighed. We observed a marked weight reduction in the miR‐502‐5p–transfected group (Figure 6D). The size of each tumour xenograft is presented (Figure 6E). To further confirm the expression of the 3 miR‐502‐5p targets, we extracted protein from the tumour xenografts and performed a Western blot assay. The results showed that all three targets were downregulated in the miR‐502‐5p–transfected group (Figure 6G). We further verified the levels of a proliferation indicator (Ki‐67) and the target genes of miR‐502‐5p in both groups through immunohistochemical experiments, and the results indicated that these proteins were consistently suppressed in the miR‐502‐5p–transfected group (Figures 6F and S4D). In conclusion, the in vivo tumour xenograft experiment further confirmed the tumour suppressor role of miR‐502‐5p in BCa. Consequently, to generalize the main findings in our study, all the processes mediated by miR‐502‐5p were involved in a schematic diagram (Figure 6H).

## 4 DISCUSSION

Recent evidence has revealed that downregulation of miRNAs is closely related to the occurrence and development of numerous human diseases, especially human cancers. Studies on tumour‐related miRNAs support the notion that circulating miRNAs may serve as noninvasive biomarkers to determine the risk of cancer development, response to treatment and prognosis.^28^

The functions of miR‐502‐5p in several types of cancers have been examined, and several genes directly targeted by miR‐502‐5p, such as TRAF2, IRF‐1, SET8 and TP53, have been found.^16^, ^19^, ^29^, ^30^ Most studies suggest that miR‐502‐5p is a tumour‐inhibiting factor that enhances apoptosis and inhibits the proliferation of tumour cells. However, no information has been observed concerning the specific role and mechanism of miR‐502‐5p in BCa. In our study, we observed that the expression of miR‐502‐5p in BCa cell lines and clinical tissues was significantly downregulated due to epigenetic mechanisms. We found that the overexpression of miR‐502‐5p significantly inhibited proliferation and migration and induced apoptosis in BCa cells. Both in vitro and in vivo experiments revealed the tumour suppressor role of miR‐502‐5p in bladder cancer. Furthermore, we investigated the downstream mechanisms of miR‐502‐5p in bladder cancer. After the overexpression of miR‐502‐5p, we observed significant decreases in the migration and proliferation abilities of BCa by regulating EMT and cell cycle pathways. In addition, miR‐502‐5p could induce cell apoptosis by regulating the levels of Caspase 3 and Parp‐1.

Three new targets of miR‐502‐5p named CCND1, DNMT3B and NOP14 were ascertained and verified in our experiments. CCND1, belonging to the highly conserved cyclin family, was identified as a regulator of the cell cycle. This protein forms a complex with CDK4 and CDK6, which are responsible for cell cycle G1/S transition.^31^ A previous study identified that CCND1 was upregulated in BCa.^32^ In addition, microRNAs, including miR‐16 and miR‐576‐3p, that inhibit cell proliferation by targeting CCND1 in BCa have been identified.^9^, ^33^ In our research, we found that miR‐502‐5p could regulate cell proliferation by targeting CCND1 in BCa. NOP14 nucleolar protein (NOP14) is frequently identified as a regulator involved in pre‐18s rRNA processing and small ribosomal subunit assembly.^34^ A previous study demonstrated that NOP14 enhances the stability of mutp53, enabling mutp53 to participate in the progression of pancreatic cancer.^35^ NOP14 could suppress cancer metastasis by regulating the Wnt/β‐catenin signalling pathway in cancers.^36^, ^37^ In our research, we found that NOP14 is involved in cell proliferation and migration and induces apoptosis in BCa cells. Furthermore, NOP14 silencing reduced the expression of cell cycle‐related proteins and MMP9 and upregulated the expression of cleaved Caspase 3.

DNA methyltransferase 3 beta (DNMT3B) is a DNA methyltransferase that is considered responsible for the process of CpG methylation.^38^ The transcriptional repression mediated by DNA methylation at CpG island‐associated promoters could lead to gene silencing, which is the representative activity described for DNMT3B. A previous study revealed that the inactivation of tumour suppressor genes resulting from epigenetic silencing associated with hypermethylation is crucial to the development of human cancer.^39^ In our study, we found that the hypermethylation of E‐cadherin promoters mediated by DNMT3B induced cell migration in BCa cell lines. In addition, DNMT3B silencing upregulated the expression of p15, which may result from the hypermethylation of p15 promotors. These results revealed the mechanism of DNMT3B mediated by miR‐502‐5p in regulating EMT progression and the cell cycle pathway in BCa.

In conclusion, we report the following findings. Overexpression of miR‐502‐5p markedly inhibited cell proliferation via G1 phase arrest and induced apoptosis and migration via the EMT pathway. CCND1, NOP14 and DNMT3B are direct downstream targets of miR‐502‐5p. Silencing of NOP14 markedly inhibited cell migration and proliferation. Silencing of DNMT3B significantly inhibited cell migration via methylated E‐cadherin promotors and cell proliferation. In addition, animal studies have confirmed the tumour‐suppressing role of miR‐502‐5p in vivo. Our studies identified the crucial role of miR‐502‐5p in BCa progression, and we expect that our findings on the miR‐502‐5p–mediated regulatory network propose a new tumour biology mechanism and will provide potential treatments for BCa targeting in the future.

## CONFLICT OF INTEREST

The authors declare no conflicts in this study.

## AUTHOR CONTRIBUTIONS

B.Liu, X.Wang, X.Zheng and L.Xie designed research; Y.Ying, J.Li, H.Xie and H.Yan performed experiments; K.Jin, L.He and J.Wu analysed data; X.Ma, X.Xu and J.Fang edited the report for intellectual content; Y.Ying wrote the report, and all authors read and approved the final report.

## Data Availability

All data generated or analysed during this study are included in this article.
