# Supplementary figures and images for: CCND1, NOP14 and DNMT3B are involved in miR‐502‐5p–mediated inhibition of cell migration and proliferation in bladder cancer
Source: Cell Prolif. 2020 Jan 23;53(2):e12751. doi: 10.1111/cpr.12751 (PMC7048215; doi:10.1111/cpr.12751)

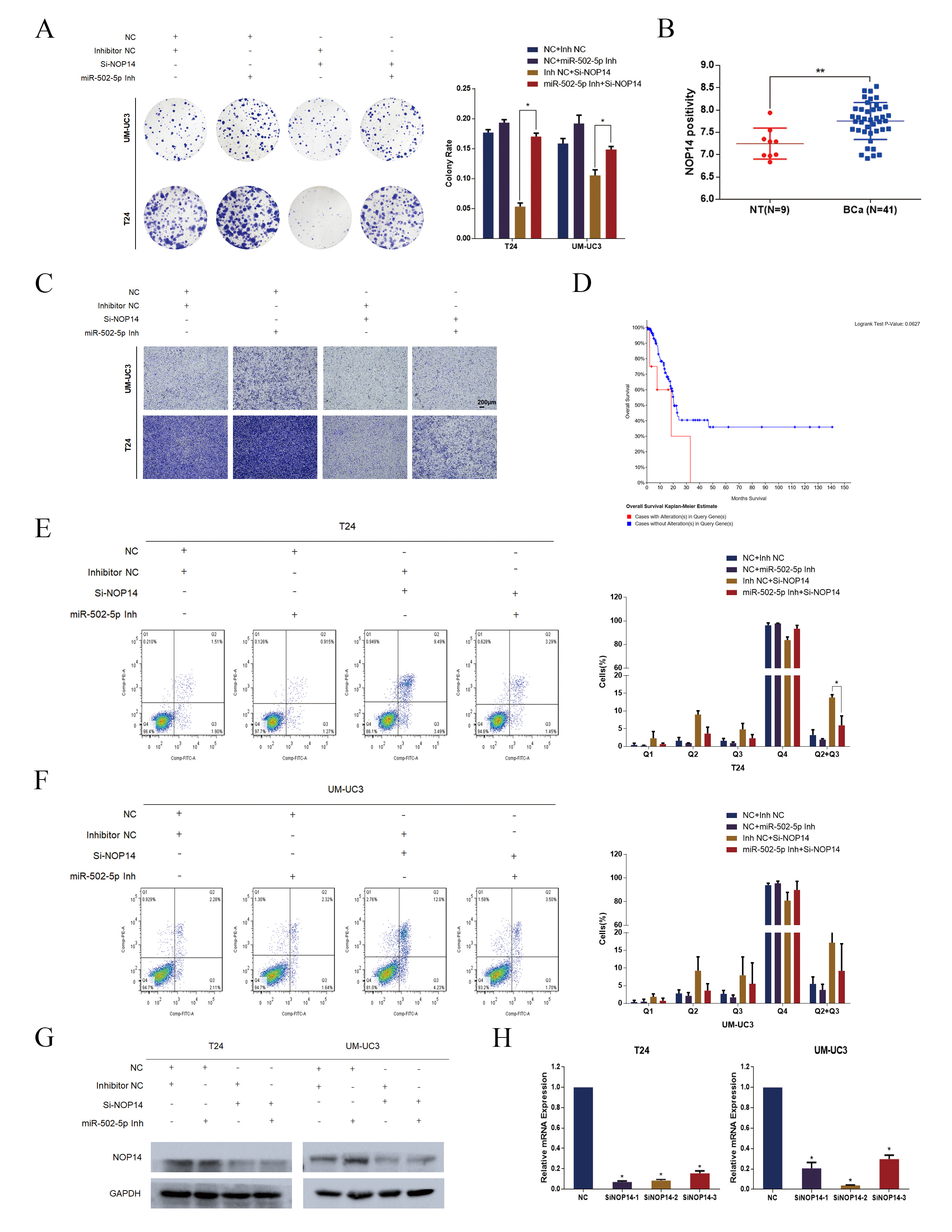

Supplement: Supplementary file 1 [file CPR-53-e12751-s001.jpg]

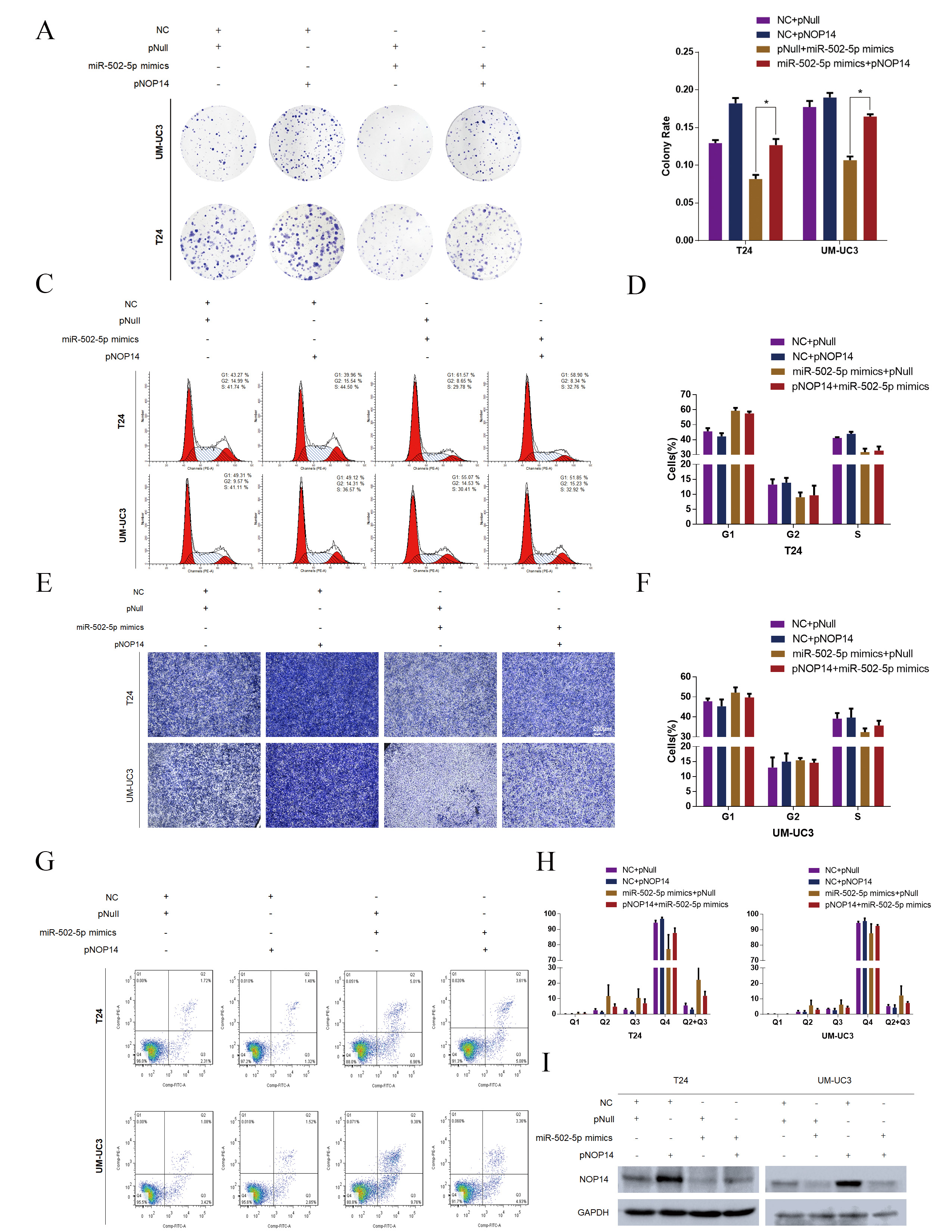

Supplement: Supplementary file 2 [file CPR-53-e12751-s002.jpg]

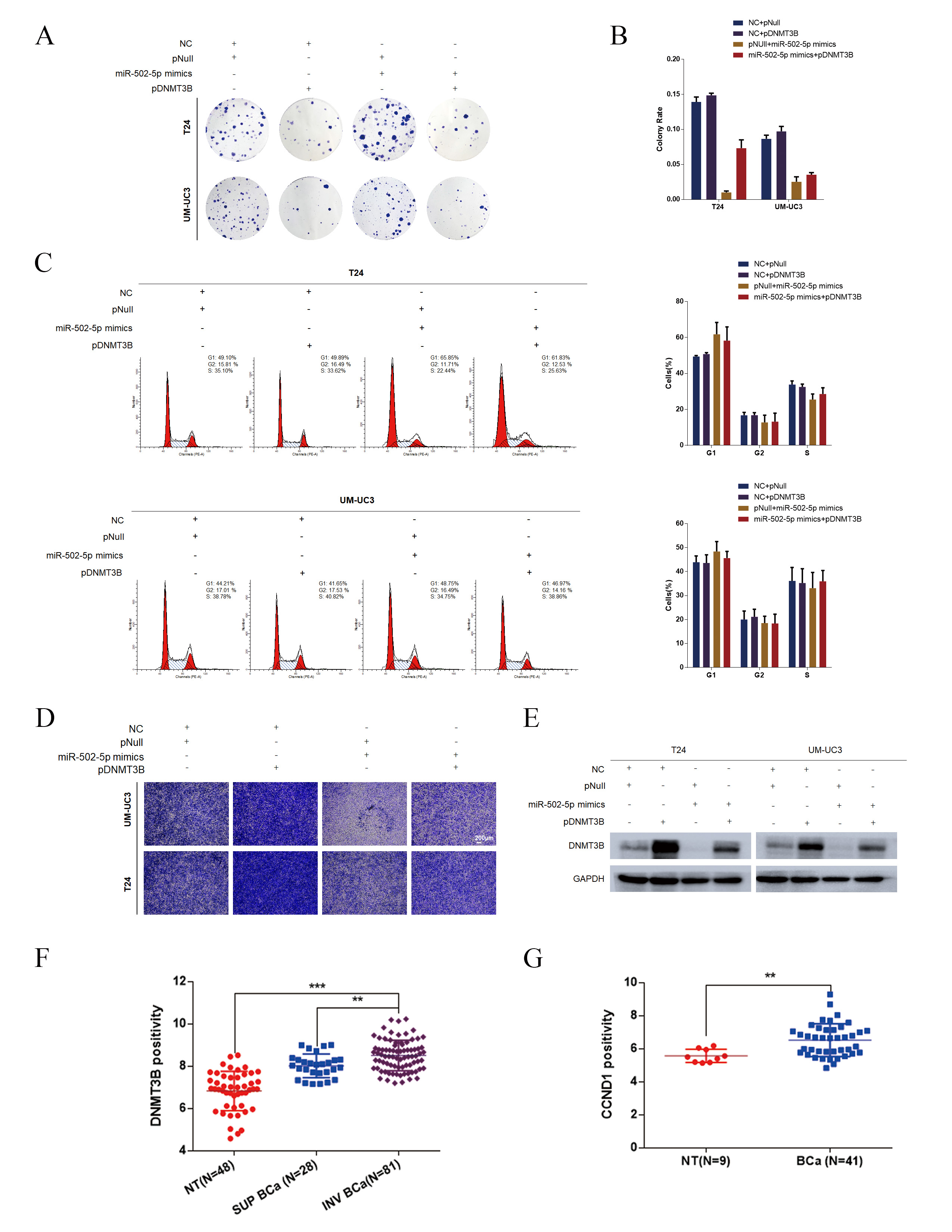

Supplement: Supplementary file 3 [file CPR-53-e12751-s003.jpg]

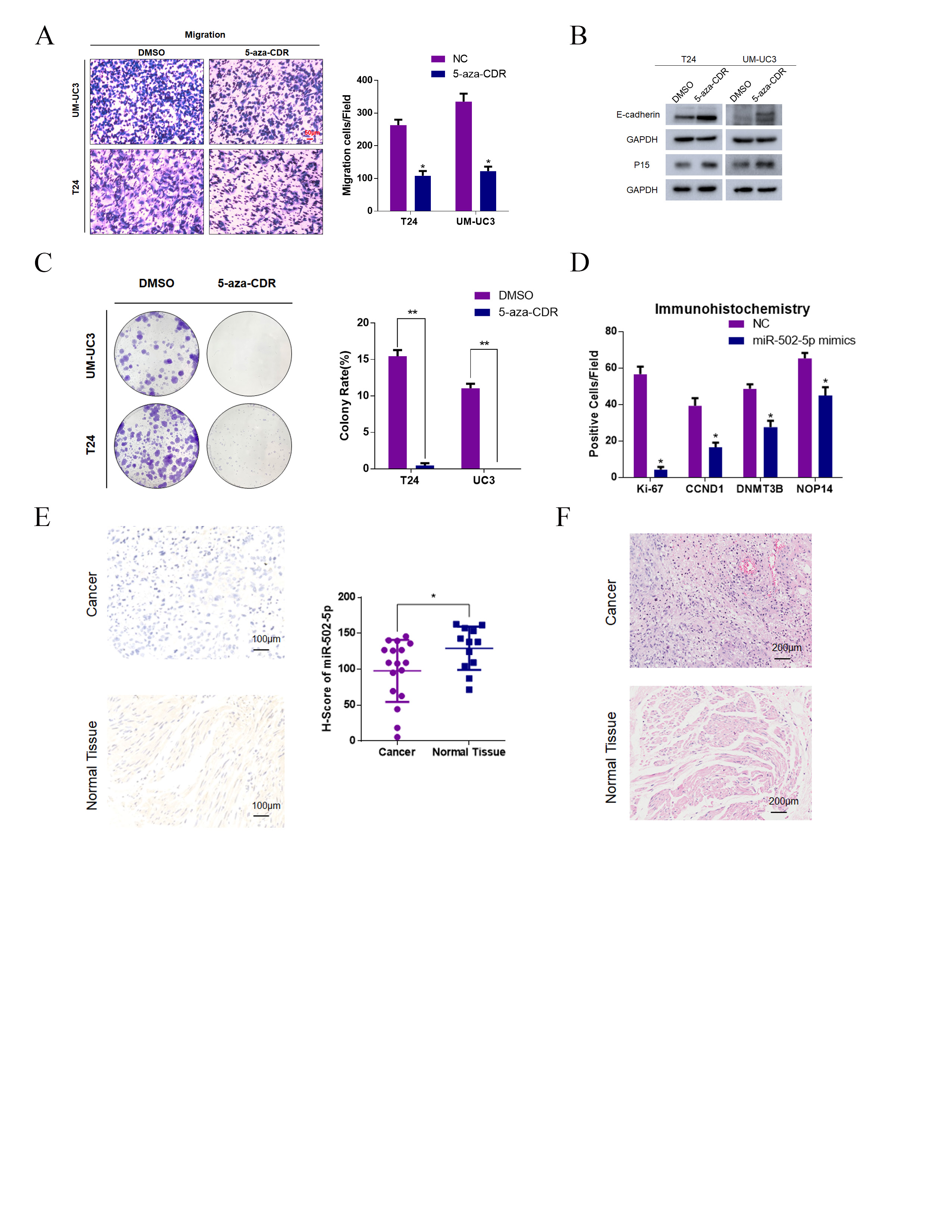

Supplement: Supplementary file 4 [file CPR-53-e12751-s004.jpg]
