# Supplementary material for: CCND1, NOP14 and DNMT3B are involved in miR‐502‐5p–mediated inhibition of cell migration and proliferation in bladder cancer
Source: Cell Prolif. 2020 Jan 23;53(2):e12751. doi: 10.1111/cpr.12751 (PMC7048215; doi:10.1111/cpr.12751)
Supplement: Supplementary file 5 [file CPR-53-e12751-s005.docx]

Supplementary Table 1.The oligonucleotides used in this study.

|  | |
| --- | --- |
| **Name^a^** | **Sequence(5’->3’)^b^** |
| miR-502-5p mimics (sense) | ATCCTTGCTATCTGGGTGCTA |
| NC (sense) | ACTACTGAGTGACAGTAGA |
| miR-148a-3p-Inh (sense) | TAGCACCCAGATAGCAAGGAT |
| miR-502-5p F | ATCCTTGCTATCTGGGTGCTA |
| U6 F | TGCGGGTGCTCGCTTCGGCAGC |
| GAPDH F | AAGGTGAAGGTCGGAGTCA |
| GAPDH R | GGAAGATGGTGATGGGATTT |
| SP1-F | TGGCAGCAGTACCAATGGC |
| SP1-R | CCAGGTAGTCCTGTCAGAACTT |
| DNMT3B-F | AGGGAAGACTCGATCCTCGTC |
| DNMT3B-R | GTGTGTAGCTTAGCAGACTGG |
| CCND1-F | GCTGCGAAGTGGAAACCATC |
| CCND1-R | CCTCCTTCTGCACACATTTGAA |
| NOP14-F | GAAGGCCAACTCCAATCCGTT |
| NOP14-R | AAGTCTGTGTACGCTTCCTGA |
| SET8-F | ACCGACGGGGAGAACGTATT |
| SET8-R | GCATTCCAGAGCATTTGTTCG |
| EGFR-F | AGGCACGAGTAACAAGCTCAC |
| EGFR-R | ATGAGGACATAACCAGCCACC |
| ZEB2-F | CAAGAGGCGCAAACAAGCC |
| ZEB2-R | GGTTGGCAATACCGTCATCC |
| CDK6-F | GCTGACCAGCAGTACGAATG |
| CDK6-R | GCACACATCAAACAACCTGACC |
| MET-F | AGCAATGGGGAGTGTAAAGAGG |
| MET-R | CCCAGTCTTGTACTCAGCAAC |
| EEF1A1-F | TGTCGTCATTGGACACGTAGA |
| EEF1A1-R | ACGCTCAGCTTTCAGTTTATCC |
| Si-NOP14 (sense) | CCAGUGACCCUGAGAGCAATT |
|  | GGUUUAUACCUGAGCUUAUTT |
|  | GGAAGAGGCUGAUCCACAATT |
| Si-DNMT3B (sense) | GUACCAUGCUCUGGAGAAATT |
|  | CCUCAAGACAAAUUGCUAUTT |
|  | GGAUGUUUGAGAAUGUUGUTT |
| NOP14-wt-F | cTACATTTTATAAATAAGGCAAGGAACTGGACATTACCTCACAg |
| NOP14-wt-R | tcgacTGTGAGGTAATGTCCAGTTCCTTGCCTTATTTATAAAATGTAgagct |
| NOP14-mut-F | cTACATTTTATAAATAAGCGTTCCTACTGGACATTACCTCACAg |
| NOP14-mut-R | tcgacTGTGAGGTAATGTCCAGTAGGAACGCTTATTTATAAAATGTAgagct |
| CCND1-wt-F | cGATTGGACAGGCATGGGTGCAAGGAAAATTAGGGTACTCAg |
| CCND1-wt-R | tcgacTGAGTACCCTAATTTTCCTTGCACCCATGCCTGTCCAATCgagct |
| CCND1-mut-F | cGATTGGACAGGCATGGGTCGTTCCTAAATTAGGGTACTCAg |
| CCND1-mut-R | tcgacTGAGTACCCTAATTTAGGAACGACCCATGCCTGTCCAATCgagct |
| DNMT3B-wt-F | cAGGAGAGGAGTGTGAAGCAAGGAGCTTAGATAAGACACCCg |
| DNMT3B-wt-R | tcgacGGGTGTCTTATCTAAGCTCCTTGCTTCACACTCCTCTCCTgagct |
| DNMT3B-mut-F | cAGGAGAGGAGTGTGAACGTTCCTGCTTAGATAAGACACCCg |
| DNMT3B-mut-R | tcgacGGGTGTCTTATCTAAGCAGGAACGTTCACACTCCTCTCCTgagct |
|  |  |
| ^a^ F, forward primer; R, reverse primer.  ^b^ Restriction sites are in bold | |
