# Supplementary material for: CCND1, NOP14 and DNMT3B are involved in miR‐502‐5p–mediated inhibition of cell migration and proliferation in bladder cancer
Source: Cell Prolif. 2020 Jan 23;53(2):e12751. doi: 10.1111/cpr.12751 (PMC7048215; doi:10.1111/cpr.12751)
Supplement: Supplementary file 6 [file CPR-53-e12751-s006.docx]

| Supplemental Table 2. Clinical data of the patients. | | | | |
| --- | --- | --- | --- | --- |
| Patient no. | Sex | Age | TNM stage | Histological grade |
| 1 | Male | 62 | T2N0M0 | III |
| 2 | Male | 60 | T1N0M0 | I |
| 3 | Male | 53 | T1N0M0 | III |
| 4 | Male | 86 | T1N0M0 | III |
| 5 | Male | 55 | T1N0M0 | II |
| 6 | Female | 74 | T2N0M0 | III |
| 7 | Male | 56 | T2N0M0 | III |
| 8 | Female | 76 | T3N0M0 | III |
| 9 | Male | 65 | T2N0M0 | II |
| 10 | Male | 76 | T3N0M0 | III |
